# Clinical Strategies for Identifying Pediatric Patients with Tuberculosis at Risk of Developing Depressive Disorders

**DOI:** 10.3390/clinpract14060187

**Published:** 2024-11-05

**Authors:** Oana Mariana Mihailov, Anamaria Ciubară, Valerii Luțenco, George Țocu, Loredana Stavăr Matei, Raul Mihailov

**Affiliations:** 1 “Sfântul Spiridon” Pneumophthisiology of Galati, 800189 Galați, Romania; oana.mihailov@ugal.ro; 2 Faculty of Medicine and Pharmacy, “Dunărea de Jos” University of Galati, 800216 Galați, Romania; anamaria.ciubara@ugal.ro (A.C.); loredana.matei@ugal.ro (L.S.M.); raul.mihailov@ugal.ro (R.M.); 3 Emergency Hospital “Sf. Ap. Andrei”, 800578 Galați, Romania; valerii.lutenco@ugal.ro

**Keywords:** depressive disorders, tuberculosis, predictive score, children

## Abstract

Background: Tuberculosis remains a major global public health problem, affecting millions of people every year, including children. At the same time, depressive disorders are among the most common mental disorders in children and adolescents, significantly influencing their quality of life and development. The intersection between these two pathologies—tuberculosis and depressive disorders—in pediatric patients raises complex clinical challenges that require effective identification and intervention strategies. Materials and Methods: A total sample of 190 patients aged between 7 and 18 years who presented to Galati “St. Spiridon” Pneumophthisiology Hospital between January 2019 and December 2021 was used. Objective: The main objective of this paper is to achieve a predictive score of depressive disorders in pediatric patients diagnosed with a form of tuberculosis. This score is particularly important because it helps to identify and treat early depressive disorders in children previously diagnosed with tuberculosis, resulting in increased compliance with anti-tuberculosis treatment, decreased dropout rate, and an optimal duration of hospitalization and surveillance, which positively influences the incidence of tuberculosis. Results: The final score is determined by a rating of a total of 9 points: if the value is below 4 points, there is a minor risk of affective disorders; if the value is between 4 and 6 points, there is a medium risk of affective disorders; if the value is above 6 points, there is a severe risk of affective disorders. Conclusions: A detailed clinical assessment, the usage of screening tools, long-term monitoring, multidisciplinary interventions, and family support are essential to ensure an effective management and to improve the life quality of these children.

## 1. Introduction

Tuberculosis (TB) remains a major global public health problem, affecting millions of people every year, including children [1].

Risk factors, such as poor socio-economic conditions and limited access to medical care, contribute to the spread of the disease and its worsening impact on children’s mental health [2].

At the same time, depressive disorders are among the most common mental disorders in children and adolescents, significantly influencing their quality of life and development. The intersection between these two pathologies—TB and depressive disorders—in pediatric patients raises complex clinical challenges that require effective identification and intervention strategies [1].

In Romania, depressive and anxiety disorders in children and adolescents are a growing problem, and the statistics present a worrying picture. According to UNICEF, approximately 33% of adolescents aged 11–15 feel sad several times a week, which is more than double the international average (13% across 45 countries). Additionally, 21.5% of Romanian youths reported feeling depressed in the last six months [3].

In recent years, depression cases have increased dramatically among children aged 11–14, and the pandemic, together with the vulnerable social climate, have exacerbated these issues, and in this situation children need therapeutic support to manage the resulting traumas [4]. In addition, it is noteworthy that, among adolescents over 15 years old, the prevalence of depressive episodes is much higher, reaching 69% [3].

Globally, depression significantly affects children and adolescents. According to the World Health Organization (WHO), approximately 280 million people worldwide suffer from depression, which represents about 3.8% of the global population [5]. Children and adolescents are not immune to this problem, and depression can start at a very young age [6]. Estimates show that the prevalence of depression in children varies between 2 and 4%, depending on the region and age group [5].

A report by the Institute for Health Metrics and Evaluation (IHME) shows that depressive disorders are more common in regions with low and middle incomes, where access to treatment is extremely limited. In many areas, more than 75% of children and adolescents suffering from depressive disorders do not receive the treatment they need due to the lack of specialized services, social stigma, and low investment in mental health [6].

Additionally, depression is a global mental health issue that affects more women than men, and the prevalence among adolescents and young people can vary from 5% to 10% in certain regions [5].

At the global level, addressing depressive disorders in children involves various interventions, including prevention, early diagnosis, and integrated treatment. One of the main strategies is the Early Adolescent Skills for Emotions (EASE) program, developed by the World Health Organization (WHO) and UNICEF, targeting children aged 10–15. This program is based on Cognitive Behavioral Therapy (CBT) techniques, including seven group sessions for adolescents and three sessions for parents, and is currently implemented in countries such as Jordan, Pakistan, Lebanon, and Tanzania. The EASE program is designed to be delivered by non-specialized personnel who are trained and supervised by mental health professionals, making it accessible in resource-limited areas [7].

Another innovative approach includes the use of Ecological Momentary Assessment (EMA), which utilizes mobile devices to monitor adolescents’ emotional states in real time. This can be integrated with other therapies (e.g., CBT) to obtain a clearer picture of the factors triggering depressive episodes and to adapt interventions according to the specific needs of each individual [8].

Tuberculosis is caused by the Mycobacterium tuberculosis bacteria and is air-borne [1]. It affects the lungs, but can also involve other organs, complicating the diagnosis and treatment. In children, the developing immune system makes them more vulnerable to severe infections and the spread of the disease [9,10].

At the same time, children suffering from chronic diseases such as TB are at greater risk of developing depressive disorders due to the multiple challenges they are facing: physical pain, social isolation, and its impact on education and daily life [11].

The social stigma associated with tuberculosis can worsen depressive symptoms in children affected by this disease. The stigma can lead to social isolation and feelings of shame, thus affecting children’s mental health. It is essential that interventions also address these issues in order to reduce the negative impact of stigma [12].

Depressive disorders in children suffering from tuberculosis in Romania pose a significant concern, primarily due to the psychological burden of the disease and the stigma it entails. Tuberculosis, an infectious disease predominantly affecting the lungs, can also present in extrapulmonary forms, impacting various organs. Children diagnosed with TB may experience a range of symptoms, including persistent fever, chronic fatigue, irritability, and developmental delays. The chronic nature of the disease and its associated symptoms have a detrimental effect on their mental health, elevating the risk of developing depression and anxiety. Such psychological distress is further exacerbated by the social isolation and stigmatization that often accompany the diagnosis, contributing to a higher prevalence of emotional disorders in this vulnerable group. Unfortunately, the prevalence of depressive disorders in children with tuberculosis is not well-documented in Romania, but the risk is high due to the long-term impact of the disease on physical health and quality of life. Children with tuberculosis may face stigma and social isolation, exacerbating depressive symptoms and contributing to the development of complex emotional disorders.

Managing these cases requires a multidisciplinary approach that includes not only specific medical treatment for tuberculosis but also psychological support and psychoeducation for both the child and the family. Mental health interventions, such as cognitive behavioral therapy and counseling programs, are essential to help children cope with stress and improve their long-term mental health [5,13].

In conclusion, addressing depressive disorders in children with tuberculosis in Romania must be integrated, including both the treatment of the underlying disease and mental health interventions. Increasing access to counseling services and reducing the associated stigma are essential to improve the quality of life for these children.

Understanding the link between TB and depressive disorder is crucial for developing identification and intervention strategies. Research suggests that there is a two-way link between chronic infectious diseases and mental disorders. On the one hand, TB infection and its treatment can cause depressive symptoms through chronic stress, medication side effects, and social isolation. On the other hand, depression can affect the adherence to TB treatment and, implicitly, the prognosis of the disease [14].

The objective of this study is to analyze and investigate the clinical methods and tools used to identify specific risk factors and to develop appropriate interventions for preventing and treating depression in tuberculosis patients. The study aims to examine the available data, evaluate the findings from other studies, and formulate evidence-based recommendations to improve the management of these patients.

This article explores the clinical strategies for identifying pediatric TB patients who are at risk of developing depressive disorders.

## 2. Materials and Methods

1. Study Population

The study was a prospective study conducted over a three-year period, from January 2019 to December 2021. The sample included a total of 190 patients, aged between 7 and 18 years, diagnosed with primary tuberculosis, secondary tuberculosis, or tuberculous pleurisy. These patients presented at the “Sf. Spiridon” Pulmonology Hospital in Galați and the Tuberculosis Dispensaries in Galați, Tecuci, and Târgu Bujor. Each patient was enrolled in the study and had a personal follow-up file where relevant socio-demographic, clinical, and paraclinical data were recorded.

The patients received tuberculostatic or chemoprophylactic treatment for at least 6 months, according to national and international tuberculosis treatment protocols. Clinical and paraclinical evaluations were carried out either at the time of hospital admission or upon presentation at the tuberculosis dispensaries in order to monitor disease progression and response to treatment. The prospective design of the study allowed for the continuous follow-up of the patients throughout the study period, providing valuable information on treatment efficacy and the evolution of the condition over time.

2. Inclusion and Exclusion Criteria

The study included pediatric patients aged between 7 and 18 years. Exclusion from the study was based on the following criteria:

- Age over 18 years;
- Refusal to participate in the study or to provide the necessary information (including refusal to complete the questionnaires);
- Patients declared deceased during the study.

3. Ethical Considerations

Data collection and processing were carried out with respect to patient anonymity. Approval was obtained from the Bioethics Committee of the Galați Pulmonology Hospital to access and collect personal information from the hospital’s database (observation files, consultation registers, imaging evaluation records). Consent was obtained under the following conditions:

- From the patients’ legal representatives by completing the provided questionnaire;
- From the management of the medical units for the use of the archives;
- Consents were collected in accordance with the current legislation of the World Health Organization (WHO) and the European Union regarding research on human subjects in the medical field, in compliance with the latest version of the Declaration of Helsinki.

4. Clinical and Psychological Evaluation

The evaluation included the completion of preliminary questionnaires and their reassessment at established intervals (initial assessment at admission or at presentation in the dispensaries when the diagnosis was established, followed by a reassessment at 3 months). The initial evaluation (T0) included socio-demographic, clinical, and paraclinical data, as well as preliminary questionnaires for identifying the risk of depression. The reassessment at T1 included repeating the questionnaires and analyzing the clinical, paraclinical, and psychological evolution of the patients.

5. Questionnaires Used

Table 1 presents the individual questionnaire used in the patient’s anamnesis, including the 10 questions applied, which are synchronized with the points later addressed in the CDI questionnaire [15].

The average time for administering the questionnaires was approximately 15 min.

The Children’s Depression Inventory (CDI) was used to assess the presence and severity of depressive symptoms in children and adolescents. CDI was created by Maria Kovacs and consists of two self-report forms: a long version comprising 27 items and a short version with only 10 items.

The shorter version, CDI:S, was used for a quick assessment of depressive symptoms. CDI measures five main factors: negative mood, interpersonal problems, ineffectiveness, anhedonia, and low self-esteem.

6. Description of the CDI Scale [15].

The CDI has a scale (Table 2) ranging from 0 to 54 points. To differentiate between children with and without depressive issues, specific thresholds were established: a score of 13 for clinical samples and a score of 19–20 for non-clinical states. Thus, CDI values are used to identify depressive potential in children.

7. Paraclinical Investigations

Paraclinical investigations were conducted within the Medical Analysis Laboratory of the Galați Pulmonology Hospital. Hematological investigations were performed using the Celltac analyzer (Nihon Koden, Japan), and biochemical investigations were carried out using the Vitros 4600 analyzer. Imaging investigations were performed using the DRGEM device, GXR-SD.

Hematological Investigations: Performed using the Celltac analyzer (Nihon Koden, Japan), which measures 16 parameters. Hemoglobin levels were determined spectrophotometrically, providing an accurate overview of the patients’ hematological status.

- Biochemical Investigations: Conducted using the Vitros 4600 analyzer to evaluate liver function and serum levels of inflammation and nutrition markers, providing critical information about the patients’ overall health status.
- Imaging Investigations: X-rays were performed using the DRGEM, GXR-SD device to confirm the diagnosis and monitor the treatment response.

These laboratory investigations were used to complement the clinical and psychological assessments of the patients, providing a more detailed perspective on their general health status and the impact of tuberculosis on their physical and psychological condition.

The paraclinical investigations in the study were carried out both at the time of patient admission and during their follow-up at the TB dispensaries. These data were collected directly from clinical evaluations as well as from the patients’ medical records, ensuring the comprehensive monitoring of each patient. Laboratory tests performed as part of the paraclinical investigations included complete blood count, ESR (erythrocyte sedimentation rate), liver function tests, and microbiological examinations such as sputum smear microscopy and culture for Mycobacterium tuberculosis. Additionally, imaging techniques like chest X-rays were utilized to assess lung involvement.

8. Statistical analysis

The authors have included a detailed assessment of the studied data, using a methodological approach based on descriptive and analytical statistics to interpret the obtained results. The analysis was conducted using the SPSS IBM Statistics V26 software, where variables such as age, weight, TST (tuberculin skin test reactivity index), CDI scores, and the symptomatic evolution of the patients were included. The author employed various statistical methods, including a histogram for checking the distribution of the series and Pearson correlation.

The findings were presented through synoptic tables and graphs that synthesized the collected information. The author’s evaluation focused on identifying correlations between socio-demographic variables (gender, age, environment of origin) and the risk of developing affective disorders in pediatric patients with tuberculosis. In addition, the analysis highlighted the differences between risk groups, contributing to the formulation of working hypotheses regarding the factors that could influence the evolution of depressive symptoms.

This evaluation was designed to establish individualized risk profiles and to support a multidisciplinary approach in the management of pediatric patients with tuberculosis, highlighting both predisposing and protective factors.

## 3. Results

After corroborating the distribution according to the environment of origin and gender of the subjects, it was observed that in the rural area, the number of male subjects was the majority (40.10%, n = 77), while only 30.73% (n = 59) were female. On the other hand, in the urban area there was a symmetrical distribution between the two genders, 14.74% (n = 28) being male, and 14.74% (n = 28) being female.

Following the correlation of the age groups with the environment of origin, it was observed that in rural areas most of the subjects were aged between 15 and 18 years (36.32%, n = 69), 18.95% (n = 36) between 11 and 14 years, and 15.26% (n = 29) were aged between 7 and 10 years. On the other hand, the distribution in the urban area of the subjects according to the age group showed a quasi-symmetry between the age groups 15–18 and 11–14 years, representing 13.16% (n = 25) and 10.53% (n = 20) of the studied group, respectively. Subjects aged 7 to 10 years from urban areas accounted for the lowest percentage of the study group (5.79%, n = 11). These details can be found in Table 3.

The analysis of the types of tuberculosis pathology diagnosed within the study group revealed that the majority of patients, approximately 82% (Table 4), were diagnosed with occult primary tuberculosis. On the other hand, tuberculous pleurisy proved to be the rarest form of tuberculosis identified in this group, affecting only 2.11% of the patients. This suggests that pleural involvement in tuberculosis manifestations is relatively uncommon in this clinical context.

To present the incidence of decisions to perform the CDI test based on various socio-demographic characteristics, such as place of origin, distance from medical services, gender, and age group, a contingency table (Table 5) was created. This table reflects the clinical significance of the CDI test at the evaluation moments T0 (initial) and T1 (reevaluation), providing an overview of the suspicion of the onset of depressive disorders and the decisions to undertake medical interventions. Additionally, it highlights the differences between cases where there is no suspicion of depressive disorders and those where such suspicions are identified, both at T0 and T1.

Thus, regarding the decision to perform the CDI (in the 158 subjects, with the consecutive exclusion of a number of 32 patients who did not attend the reassessment), the following details can be observed, which reinforce the management algorithm:

✧ It was performed mainly for the subjects from rural areas (n–110), with a percentage difference of 39.24% compared to the subgroup of patients from urban areas. This is partly justifiable starting from the prerequisite that patients from rural areas do not have such easy access to medical services (which is why their poor follow-up can “lose sight” of a number of clinical signs indicating the onset of depressive disorders. It is also known that the rural environment, due to the small community of people, can contribute to the desire for social isolation, due to the stigma associated with a diagnosis of this type. Over time, this social isolation, combined with the loss of activities typical of the rural area (working in the fields, caring for animals, etc.) will accentuate the spectrum of affective disorders.

The CDI was mostly performed in patients who lived at a distance of less than 10 km from the follow-up health unit. Although there is no statistical significance regarding the interaction of these two variables, a working hypothesis according to which the subjects’ monitoring was more difficult due to the fact that the duration of the study included the period of the SARS-CoV-2 pandemic (respectively, the state of emergency) can be issued.

The quasi-symmetric distribution of the decisions to perform the CDI depends on the gender of the patients (with a slight predominance among male subjects, n = 84).

The age groups of the patients represent a variable that determines an extremely strong significance (at both ends) from a statistical point of view, with the decision to perform the CDI. Thus, the existence of a distribution characterized by an upward slope, dependent on the ages of the subjects, with the consecutive predominance of the CDI performed in those aged 15–18 years (n = 67), is observed.

The clinical significance of the CDI and the interpretation of the results obtained by patients presents statistically significant distributions; these are also qualitatively significant, with a series of parameters analyzed as follows:

✧ A 36.98% percentage difference indicating the association of the CDI values corresponding to scores with clinical significance of affective disorders with the rural environment.
✧ The predominance (n = 45) of clinical significance in subjects residing less than 10 km from the medical care facility.
✧ Mostly, the male pediatric patients have CDI scores with clinical significance (n = 80).

At the time of T0, the predominance of affirmative answers was noted in a proportion of 90% which determined a series of subsequent implications, among which we mention the following:

✧ There is a hypothesis that it is not the mere exposure of the pulmonary TB diagnosis in the pediatric patient that determines the depressive disorder (by being directly and solely responsible).
✧ Often, it is noted that the isolation/self-isolation of the subject (prior to the issuance of the diagnosis of certainty), the existence of associated disorders (inefficiency, insomnia, decreased appetite), and the detection of family dynamics disorders corroborate with each other and aggravate the psychological status of the patient.
✧ About 90% of young people (older children, pre-adolescents, but especially adolescents) are at risk of developing depressive disorders, often due to the individual/environmental particularities they face on a daily basis.

In the case of the questionnaire carried out at T1, a new distribution is noted, which again allows the issuance of some working hypotheses, as follows:

✧ It can be suspected that, at T1, more precisely, 3 months after the initial evaluation, some of the patients enrolled in the study group showed improvements (at least from a subjective point of view) in the depressive disorders.
✧ The decrease in the incidence of affirmative answers can also be due to an extremely simple fact: as mentioned in the literature, the simple knowledge of a diagnosis of certainty determines the subject to obtain the necessary materials for initiating healing, with the consequent improvement of the symptomatology.

From the point of view of suspicions of depressive disorders at the initial evaluation, respectively, at the T1 reevaluation, the following is highlighted:

✧ The mean age expressed in full years is 14 years, in both situations.
✧ The patients recorded an average number of five visits to the attending physician for assessment/reassessment until these suspicions were established.
✧ The mean weight of the subjects, expressed in kg, was 48 kg for those who under suspicion since performing T0, while it was 59 kg for those where T1 was also performed, respectively.
✧ The mean value of the IDR expressed in mm was 11 mm for both categories of patients.
✧ For both groups of subjects, a mean number of two cardiopulmonary radiographs were performed, until suspicion was confirmed.
✧ The oxygen saturations established in the atmospheric air maintained a mean value of 98%.
✧ The average number of items that were identified as affirmative answers, for the T0 and T1 questionnaires, is six items.
✧ The total (mean) value of the CDI score for patients who can be classified as subjects who may be at risk of later occurrence of depressive disorders was 18 points, distributed as follows:
  ♦ Negative mood scale: MV (mean value) of 4 points for both situations.
  ♦ Interpersonal problems with a MV of 2 points each.
  ♦ Inefficiency, as an indicator, recorded a mean value of 3 points.
  ♦ Anhedonia was defined by a mean value of 5 points for the subgroup of those who performed T0 and 6 points for those in the T1 category.
  ♦ Low self-esteem was defined as having a mean value of 3 points for both situations discussed above.

The table below (Table 6) continues the presentation of the descriptive statistics previously mentioned by defining the mean values of the scalar indicators that served as reference points for decisions regarding the implementation of the evaluation questionnaires (at T0, T1, and CDI time points).

**Figure 1:**
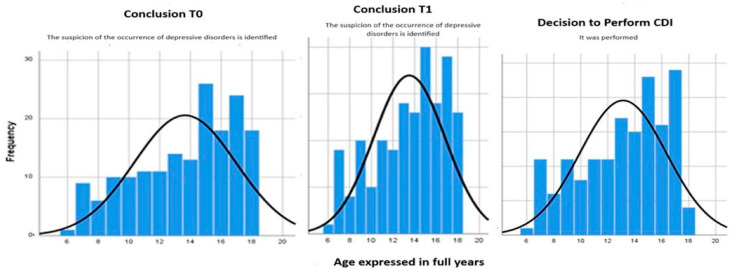
Histogram of patients’ ages at the time of initial registration, compared to the results obtained from the questionnaire at the T0, T1, and CDI evaluations.

To support the data presented in Table 6 regarding the distribution of cases for the three variables by the age factor, we created histograms that show the distribution of these values by age groups (Figure 1).

Analyzing the histograms, it can further be argued that adolescent patients (aged 15–18) are the most at risk of developing psychiatric spectrum disorders during the course of their illness. To further demonstrate the relationships between T0, T1, and CDI, as well as the factors that contribute to the development of mental disorders, a Pearson bivariate correlation was conducted for each combination (Table 7).

Personal pathological history and the reason for presentation are strongly statistically correlated with T0—the initial evaluation moment. The same strong correlation is maintained later during reevaluation (T1) and at the time of CDI. However, regarding the relationship between the decision to perform CDI and the reasons for presentation, there is no longer a strong statistical correlation (sig = 0.191).

- The decision to perform CDI is strongly influenced by the age groups of the subjects, their responsiveness (reasons for presentation), their level of cooperation (number of presentations), personal history, and, importantly, the type of tuberculosis pathology present in the subjects (sig = 0.024), as well as the degree of subject reevaluation (sig = 0.000).
- A strong correlation is also noted between the clinical significance of the data obtained from the CDI and the following factors: age groups, initial reasons for presentation, and other factors that influence the decision to perform CDI.

To evaluate the decision to administer the CDI questionnaire, considering the number of affirmative items at T0 and T1, a data summary was compiled, as presented in the table below (Table 8).

Analyzing Table 8, we can conclude that out of the 158 cases that opted to administer the CDI, the majority of patients (n = 140) presented a suspicion of developing a depressive disorder. The value of the *sig* index obtained after performing the chi-square test (conclusion T0—decision to perform the CDI) (sig = 0.155) demonstrates that there are no statistically significant differences between those who were under the suspicion of developing depressive disorders at T0 and when performing the CDI. Thus, the null hypothesis according to which detecting the suspicion of the further development of depressive disorder, at the time of taking the patient into account, does not represent a situation that necessarily indicates the need to perform/apply the pediatric depression questionnaire, and can therefore be issued at this point. The existence of an incidence of affirmative answers higher than 50% at T0 may be due in part to the association of real problems existing in the patient’s personal life, an atypical terrain, a (family or personal medical) history of psychiatric/psychological diseases, or simply due to the uncertainty of the diagnosis.

At the same time, when the statistical analysis is made from a descriptive point of view in relation to the incidents of suspecting the occurrence of depressive disorder and at the time of repeating the questionnaire, it will be observed that this time, the decision to perform the licensed depression questionnaire was taken for a total number of 158 patients. Of these, only 121 (76.58%) presented more than half of the answers coded as “yes” to the application of the individual questionnaire. This time, however, the performance of the chi-square test reveals a sig index lower than the reference value (sig = 0.000 *), which is why it can be concluded that the decision of the attending physician to perform the CDI was (statistically) dependent on the conclusion of the T1 reassessment.

The two tables presented earlier (Table 9 and Table 10) provide a numerical summary of the distribution of patients in the study group, based on the results obtained from the application of the three questionnaires. This descriptive statistic is necessary for formulating future working hypotheses as well as for performing preliminary prediction scores.

It can be observed that the majority of patients who were at risk of developing affective disorders, according to the CDI questionnaire, had approximately eight positive responses at T0 (therefore, we can infer that if we have more than the accepted value on seven items, there will be a higher risk of developing depressive disorders, making the CDI necessary) and four positive responses at T1.

The fact that the result obtained from the CDI testing appears to be correlated with the previous correlations at T0 and T1 leads us to propose the working hypothesis that a key point in the management of these patients is the stepwise evaluation of the case.

The following section will present a comparative analysis of potential symptoms related to depressive disorders (T1) in relation to a previous evaluation (T0) and various psychological and behavioral variables, such as general condition, personal relationships, perceived inefficacy, self-esteem, irritability, fatigue, insomnia, low appetite, vices, and introversion (Table 11), as well as a table comparing the two evaluation moments, T0 and T1, regarding the suspicion of depressive disorders and the decision to perform CDI, along with the interpretation of the clinical significance of CDI scores (Table 12). These two tables were introduced to demonstrate that there is, to some extent, a significant difference in psychological and behavioral variables between the two time points between evaluations (also proven by the McNemar test).

Based on the underlying synoptic tables (Table 11 and Table 12), we performed a predictive score of depressive disorders of the pediatric patient diagnosed with a form of tuberculosis. The score obtained is a benchmark for the research in question. It is the basis of the management therapy of TB patients who are at risk of developing depressive disorders.

To outline the profile of the pediatric patient diagnosed with TB, who presents the highest rate of suspected depressive disorder, we have developed a system that operates as follows: at each investigation point, the subject will be asked to complete a questionnaire that addresses ten psychological and behavioral variables. For each test, the number of positive responses will be identified. The number of positive responses for the T0 or T1 moments determines the following:

- If less than 25% of the 10 responses are positive, 0 points will be assigned.
- If 25–50% of the responses are positive, 1 point will be assigned.
- If 50–75% of the responses are positive, 2 points will be assigned.
- If over 75% of the responses are positive, 3 points will be assigned.

For the CDI test, which contains five items in total, points are assigned as follows:

- If fewer than two items are modified, 1 point will be assigned.
- If two–three items are modified, 2 points will be assigned.
- If four–five items are modified, 3 points will be assigned.

Final Score Calculation:

The three scores obtained from the tests are summed, and the total value is compared to specific thresholds:

- If the score is below 4 points, the individual will be classified as having a minor risk of affective disorders.
- If the score is between 4 and 6 points, the subject will be classified as having a medium risk of affective disorders.
- If the score is above 6 points, the individual will be considered as having a severe risk of affective disorders.
- Management Based on the Final Score:
- If a score of under 4 points is obtained, a conservative attitude with expectation will be chosen regarding psychiatric intervention.
- If the score is between 4 and 6 points, the following approaches can be chosen:
- Involvement of a psychotherapy service for specialized opinion.
- Reassessment of the CDI every month for the first three months. If the score at 1 month is lower than the initial score, the conservative approach will continue, with psychotherapy services lasting approximately 6 months, followed by a reassessment after 1 year.
- If the risk remains the same in both tests, the patient will be redirected to psychiatric services.
- If a score of over 6 points is obtained, the patient will be directly referred to psychiatric services.

With all this information at hand, preliminary hypotheses can be made as soon as the subjects are registered.

In the assessment of the risk for depressive disorders, the use of a scoring system based on patients’ responses to standardized questionnaires allows for an objective and systematic approach. This enables the precise tracking of each evaluation stage and the patient’s progress over time. Below are two examples that illustrate how the scores obtained at the initial moment (T0), at the reevaluation (T1), and based on the Children’s Depression Inventory (CDI) contribute to determining the risk level for depressive disorders. These examples highlight the importance of continuous monitoring and early intervention, depending on the severity of the symptoms.

Example 1:

Patient A.C. (initials of first and last name) responded positively to 6 out of 10 questions at T0, representing 60%, thus receiving 2 points for T0. At T1, the same patient A.C. responded positively to four questions, representing 40%, which results in 1 point for T1. For the CDI evaluation, patient A.C. responded positively to three out of five questions, earning 2 points.

Final score:

- T0: 2 points;
- T1: 1 point;
- CDI: 2 points.

The total score is 5 points, placing patient A.C. in the medium risk category for affective disorders. It is recommended to involve a psychotherapy service for a specialized opinion and to reassess the CDI monthly for the first 3 months.

Example 2:

Patient B.D. (initials of first and last name) responded positively to 8 out of 10 questions at T0, representing 80%, thus receiving 3 points for T0. At T1, the same patient B.D. responded positively to five questions, representing 50%, which results in 2 points for T1. For the CDI evaluation, patient B.D. responded positively to four out of five questions, earning 3 points.

Final score:

- T0: 3 points;
- T1: 2 points;
- CDI: 3 points.

The total score is 8 points, placing patient B.D. in the severe risk category for affective disorders. The patient will be immediately referred to psychiatric services.

## 4. Discussion

Comparing the study results with the data from the literature, a higher prevalence of depression is observed among patients with tuberculosis. Analyses conducted by Aprilia (2021) in a systematic review show that tuberculosis patients are at an increased risk of developing depression due to biological factors (such as changes in the immune system), as well as social and behavioral factors, including stigma and social isolation. It is noted that the use of selective serotonin reuptake inhibitors (SSRIs) is recommended as the first line of treatment for these patients [16].

Additionally, a longitudinal study from Ethiopia investigated the impact of depression on treatment outcomes in TB patients and concluded that depression is associated with poor treatment adherence, a higher rate of therapy discontinuation, and a lower quality of life [17]. Similarly, studies conducted in Ethiopia [18] and South Africa [19] have highlighted that the prevalence of depression is higher in rural areas and among patients with limited economic resources, which is consistent with the findings from the current study.

The findings of this study suggest that boys, particularly those from rural backgrounds, are at a higher risk of depression compared to girls, which contrasts with the existing literature that often highlights higher rates of depression among girls. Several contextual elements unique to this study may help to explain this apparent discrepancy.

One significant factor is the higher prevalence of tuberculosis among boys, leading to a greater proportion of boys participating in the study, which may have influenced the observed distribution of depression. Additionally, boys in rural areas face distinct economic and social challenges, such as limited educational opportunities and the responsibility of financially supporting their families, which can increase their risk for depression.

Cultural and gender norms also play a role in how depression is manifested and perceived. Depressive symptoms in boys may appear as irritability or aggressive behavior, which are often underdiagnosed or misinterpreted. These manifestations, combined with limited access to mental health resources in rural areas and boys’ reluctance to seek help for emotional issues, can exacerbate their mental health conditions.

These aspects could explain why, in this specific context, boys appear to be at a higher risk of depression compared to girls, highlighting the need for more nuanced analyses that take into account gender and geographic location variables.

These data support the need for a multidisciplinary approach in managing pediatric patients with tuberculosis, which should include regular assessments of mental health status and specific psychosocial interventions for high-risk groups, in line with the current literature recommendations [16].

Studies conducted in the US have revealed a significant prevalence of mental disorders among children, highlighting the need for early interventions and adequate support to prevent the development of long-term complications [20].

The early identification of pediatric TB patients who are at risk of developing depressive disorders is essential to ensure effective interventions and improve prognosis. Untreated depression in children can lead to severe complications, including suicide, poor school performance, and social relationship difficulties [3].

In addition, depression can negatively affect the adherence to TB treatment, increasing the risk of relapse and complications [21,22].

Identifying risk factors for depression in children diagnosed with tuberculosis is essential, as the presence of psychiatric comorbidities can complicate treatment and increase the risk of poor clinical outcomes. Advances in this field highlight that certain demographic and clinical characteristics significantly contribute to the risk of depression.

Depression tends to be more prevalent in cases where the disease duration is longer (over 6 months) and in advanced phases of treatment where patients face treatment side effects and frustrations related to a lack of progress [8].

The WHO has launched a five-year action plan (2023–2028) to improve care for children and adolescents affected by tuberculosis, which includes measures for the early identification of depressive symptoms and integrated psychological support interventions. The goal is to address both tuberculosis and mental health issues simultaneously, ensuring holistic and patient-centered management [23].

Thus, identifying and managing risk factors are essential to improving treatment adherence and reducing mortality among children with tuberculosis, providing them with multidisciplinary support that includes not only medical treatment but also mental health interventions.

### 4.1. Strategies for Identifying Patients at Risk

#### 4.1.1. Detailed Clinical Assessment

The first and most important strategy for identifying pediatric TB patients who are at risk of developing depressive disorders is detailed clinical assessment. This includes a complete medical history, the assessment of current symptoms, and risk factors. The physician must be attentive to subtle signs of depression, such as changes in appetite, sleep disturbances, irritability, social withdrawal, and decreased school performance [15].

The initial assessment must be comprehensive, including both the physical and psychological aspects of the child’s health. It is essential to establish a relationship of trust with the child and their family in order to obtain accurate information and facilitate discussions about emotional and psychological symptoms. A thorough assessment can reveal risk factors specific to each child and guide further interventions [24].

#### 4.1.2. Use of Questionnaires and Screening Tools

Standardized screening tools for depression, tailored for children and adolescents, are essential in the early identification of depressive symptoms. Questionnaires such as the Children’s Depression Inventory (CDI) and the Revised Children’s Anxiety and Depression Scale (RCADS) can be used to assess the severity of the symptoms and to monitor their progression. These tools are useful in detecting symptoms that can be easily overlooked in regular clinical assessment [25,26].

#### 4.1.3. Children’s Depression Inventory (CDI)

Children’s Depression Inventory (CDI) is a self-reporting tool that assesses depressive symptoms in children and adolescents aged 7 to 19 years. The CDI includes 27 questions that assess depressive symptoms over the past two weeks. These questions cover various areas, such as depressed mood, loss of interest, sleep, and appetite problems, as well as suicidal thoughts. CDI is a validated tool and is widely used in clinical practice and research [15].

#### 4.1.4. Revised Children’s Anxiety and Depression Scale (RCADS)

The Revised Children’s Anxiety and Depression Scale (RCADS) is another self-reporting tool that assesses both depressive and anxiety symptoms in children and adolescents aged 8 to 18 years. RCADS includes 47 questions that assess six distinct areas: panic disorder, generalized anxiety disorder, separation disorder, social anxiety disorder, obsessive compulsive disorder, and major depressive disorder. This tool provides a comprehensive assessment of affective and anxiety symptoms and is useful in diagnosing and monitoring mood disorders in children and adolescents [24,25,26].

#### 4.1.5. Long-Term Monitoring

The long-term monitoring of pediatric TB patients is crucial, given that depressive symptoms can occur and evolve over time. Regular visits to the doctor, regular psychological evaluations, and the close monitoring of the child’s overall health are necessary to identify any changes in the child’s emotional state. This type of monitoring allows for quick interventions tailored to the individual needs of the child [27].

Long-term monitoring also involves working with the multidisciplinary care team. This includes pediatricians, psychiatrists, psychologists, and social workers, who can provide specialized support and interventions as the child evolves. A long-term monitoring plan should include regular assessments of depressive symptoms and other risk factors, as well as adjusting treatment according to the child’s needs [28].

Recommendations for improving the care of pediatric patients with TB and risk of depressive disorders include the following:

1. Developing and implementing continuous training programs for medical professionals in the field of pediatric mental health.
2. Using standardized screening tools for the early identification of depressive symptoms in children with TB.
3. Promoting a multidisciplinary approach and collaboration between pediatricians, psychiatrists, psychologists, and social workers to ensure holistic and integrated care.
4. Educating and actively involving families in the care process to provide emotional support and facilitate the early identification of depressive symptoms.
5. Integrating technology into the care of pediatric TB patients by using mobile apps and online mental health platforms to provide ongoing support and access to therapeutic resources.
6. Developing public health policies that include dedicated psychological support programs for children affected by TB, thus ensuring that they receive the necessary care for a healthy and balanced development [29].

By implementing these strategies and recommendations, more effective and integrated care can be provided for pediatric patients with TB and at risk of depressive disorders, thus contributing to improving the quality of life and long-term prognosis of these children.

Online mental health platforms can provide access to remote counseling and therapy, allowing children and their families to receive psychological support even in areas where the access to mental health services is limited [30]. Continuing professional education programs may include specialization courses in pediatric mental health, the use of screening tools, and specific therapeutic interventions for children and adolescents [31].

Depression is common in patients with chronic medical diseases, including tuberculosis. This underlines the importance of integrated physical and mental health management to ensure a full recovery and improve patients’ quality of life [32,33].

This study has limitations, such as the following:

1. Small sample size: A small number of participants may limit the ability to generalize the results to a broader population and reduce the statistical power of the analysis.
2. Limited database: The data were collected from a single geographic region, which means the results may not be representative of other populations or regions.
3. Self-reported data: The study data were collected through self-reported questionnaires, with the risk that participants may not accurately report certain aspects, potentially introducing errors into the analysis.
4. Uncontrolled psychosocial factors: In studies involving mental health, factors such as social support, family stress, or socio-economic conditions may influence the results and cannot always be fully controlled or evaluated.
5. Lack of a control group: The absence of a control group can make it difficult to evaluate the true impact of the intervention or treatment studied, as there is no comparison group that did not receive the treatment.
6. Lack of study protocol registration prior to data collection: At the time the study was initiated, registering study protocols was not a mandatory requirement at our institution or within the clinical research field in the respective region. Nevertheless, we fully adhered to the ethical and methodological standards established at the international level.

Although the protocol was not registered before data collection, the study was conducted in compliance with international ethical regulations and received approval from the ethics committee before data collection commenced. The methodology was clearly defined and remained unchanged throughout the study.

We acknowledge the importance of registering study protocols and are committed to implementing this practice in future studies to ensure a higher level of transparency and scientific rigor.

## 5. Conclusions

The assessment of the risk for depressive disorders in pediatric patients diagnosed with tuberculosis should be conducted through a systematic approach, using the scores obtained at stages T0 and T1. The final score, based on established criteria, allows for the classification of patients according to the risk of developing depressive disorders: minor risk (below 4 points), moderate risk (4–6 points), and severe risk (above 6 points).

The objective of the study was to develop a method for the early identification of depressive disorders in pediatric patients with tuberculosis, allowing for timely and appropriate interventions. By utilizing a phased and integrated approach, including the use of screening tools such as the CDI (Children’s Depression Inventory), continuous monitoring, and multidisciplinary interventions, the study aimed to enhance case management and prevent the progression of depressive disorders.

The proposed intervention model emphasizes the importance of an integrated and multidisciplinary approach in the management of pediatric tuberculosis patients, focusing on long-term monitoring and family support. This framework aligns with the study’s objective of reducing the risks associated with mental comorbidities and improving the overall quality of life for these children.

In conclusion, the study underlines the necessity of structured risk assessment and intervention strategies that contribute to better mental health outcomes in pediatric tuberculosis patients, thus supporting increased treatment compliance and more effective management.

## Figures and Tables

**Table 1:** Individual questionnaire.

Affected general condition/negative mood
Problems in personal life/family life
Ineffectiveness (as considered by the patient = work, studying, etc.)
Low self-esteem
Irritability
Fatigue
Insomnia
Decreased appetite
Vices/substance use (alcohol, tobacco, drugs)
Introversion (if the patient is an introvert or if they can initiate/have dialogs; look into the eyes)

**Table 2:** Scale description [15].

Factorial Scale	Measured Factor
Scale A	Negative disposition
Scale B	Interpersonal problems
Scale C	Inefficiency
Scale D	Anhedonia
Scale E	Low self-esteem

**Table 3:** Socio-demographic characteristics.

Socio-Demographic Characteristics			
Background environment			
Rural		Urban	
134		56	
Sex			
Male	Female	Male	Female
75	59	28	28
Age			
7–10 years	29	7–10 years	11
11–14 years	36	11–14 years	20
15–18 years	69	15–18 years	25

**Table 4:** Type of tuberculosis pathology detected in patients enrolled in the study group.

Type of Tuberculosis Pathology Detected in Patients Enrolled in the Study Group							
Occult Primary Tuberculosis		Primary Tuberculosis Adenopathy		Secondary Tuberculosis		Tuberculous Pleurisy	
No.	%	No.	%	No.	%	No.	%
155	81.58%	10	5.26%	21	11.05%	4	2.11%

**Table 5:** The incidence rate of decisions to perform CDI/the clinical significance of the test in relation to socio-demographic characteristics, as well as the conclusions from T0 and T1.

		T0 Conclusion		T1 Conclusion			Decision to Perform CDI		The Clinical Significance of CDI		
		There Is No Suspicion of the Onset of Depressive Disorders	The Suspicion of the Onset of Depressive Disorders Is Identified	There Is No Suspicion of the Onset of Depressive Disorders	The Suspicion of the Onset of Depressive Disorders Is Identified	T1 Was Not Performed	Not Performed	Performed	Without Clinically Significant Values	The Obtained Values Are Clinically Significant	CDI Was Not Performed
Origin environment	Rural	16	118	33	93	8	24	110	10	100	24
	Urban	3	53	6	48	2	8	48	2	46	8
Distance from medical services	<100 de km	5	56	10	46	5	13	48	3	45	13
	11–30 km	3	43	13	30	3	9	37	2	35	9
	31–50 km	5	14	3	14	2	3	16	2	14	3
	51–100 km	5	38	8	35	0	6	37	3	34	6
	>100 km	1	20	5	16	0	1	20	2	18	1
Gender	Male	7	96	21	75	7	19	84	4	80	19
	Female	12	75	18	66	3	13	74	8	66	13
Age groups	7–10 years	4	36	1	29	1	3	37	3	34	3
	11–14 years	7	49	9	46	1	2	54	4	50	2
	15–18 years	9	86	20	66	8	27	67	5	62	27

**Table 6:** The incidence rate of decisions to perform CDI/the clinical significance of the test, as well as the conclusions from T0 and T1 in relation to scalar indicators.

	T0 Conclusion		T1 Conclusion			Decision to Perform CDI		The Clinical Significance of CDI		
	There Is No Suspicion of the Onset of Depressive Disorders	The Suspicion of the Onset of Depressive Disorders Is Identified	There Is No Suspicion of the Onset of Depressive Disorders	The Suspicion of the Onset of Depressive Disorders Is Identified	T1 Was Not Performed	Not Performed	Performed	Without Clinically Significant Values	The Obtained Values Are Clinically Significant	CDI Was Not Performed
	Mean	Mean	Mean	Mean	Mean	Mean	Mean	Mean	Mean	Mean
Age expressed in completed years	13	14	13	14	16	16	13	13	13	16
Number of presentations	4	5	4	5	3	4	5	5	5	4
Current weight (kg)	48	48	49	46	59	57	46	43	46	57
IDR values expressed in mm	14	11	11	11	13	13	11	9	11	13
Chest X-ray number of investigations	1	2	2	2	1	1	2	2	2	1
SaO2	98	98	98	98	98	98	98	98	98	98
Total items detected at T0	4	6	6	6	6	7	6	5	6	7
Total items detected at T1	4	5	3	6		5	5	4	5	5
Negative mood scale	4	4	4	4			4	1	4	
Interpersonal problems	2	2	2	2			2	1	2	
Inefficiency	2	3	2	3			3	1	3	
Anhedonia	6	5	5	6			5	2	6	
Low self-esteem	3	3	2	3			3	1	3	
Total score resulting from performing the CDI	17	18	15	18			18	5	19	

**Table 7:** Centralized table of Pearson correlations between the conclusions obtained from T0, T1, and CDI, and a series of individual variables analyzed within the study group.

Correlations					
		T0 Conclusion	T1 Conclusion	Decision to Perform CDI	The Clinical Significance of CDI
Distance from medical services	Pearson Correlation	−0.029	−0.082	0.129	−0.137
	Sig. (2-tailed)	0.696	0.261	0.075	0.060
	N	190	190	190	190
Origin environment/background	Pearson Correlation	0.100	0.108	0.044	0.003
	Sig. (2-tailed)	0.170	0.137	0.545	0.972
	N	190	190	190	190
Age groups	Pearson Correlation	0.031	0.072	−0.265 **	0.231 **
	Sig. (2-tailed)	0.670	0.323	0.000	0.001
	N	190	190	190	190
Gender	Pearson Correlation	−0.116	−0.038	0.047	−0.094
	Sig. (2-tailed)	0.110	0.607	0.523	0.199
	N	190	190	190	190
Number of presentations	Pearson Correlation	0.137	−0.011	0.205 **	−0.184 *
	Sig. (2-tailed)	0.060	0.879	0.004	0.011
	N	190	190	190	190
Personal pathological history	Pearson Correlation	−0.291 **	−0.148 *	0.164 *	−0.195 **
	Sig. (2-tailed)	0.000	0.042	0.024	0.007
	N	190	190	190	190
Reasons for presentation	Pearson Correlation	−0.157 *	−0.162 *	0.095	−0.152 *
	Sig. (2-tailed)	0.031	0.026	0.191	0.037
	N	190	190	190	190
Epidemiological context	Pearson Correlation	0.105	0.096	0.046	−0.009
	Sig. (2-tailed)	0.150	0.186	0.531	0.904
	N	190	190	190	190
Disease stage at the time of registration	Pearson Correlation	0.034	0.033	−0.091	0.087
	Sig. (2-tailed)	0.638	0.656	0.210	0.234
	N	190	190	190	190
Type of TB pathology detected in patients enrolled in the study group	Pearson Correlation	0.033	−0.133	0.163 *	−0.174 *
	Sig. (2-tailed)	0.656	0.068	0.024	0.016
	N	190	190	190	190
Patient reevaluation	Pearson Correlation	0.138	0.229 **	−0.713 **	0.610 **
	Sig. (2-tailed)	0.057	0.001	0.000	0.000
	N	190	190	190	190

*. Correlation is significant at the 0.05 level (2-tailed). **. Correlation is significant at the 0.01 level (2-tailed).

**Table 8:** Summary table of CDI administration decisions, depending on the results obtained by patients after completing the T0 and T1 questionnaires.

		Decision to Administer CDI		Chi-Square Test with sig. Index
		Not Administered	Administered	
T0 Conclusion	No suspicion of depressive disorders	1	18	0.155
	Suspicion of depressive disorders identified	31	140	
T1 Conclusion	No suspicion of depressive disorders	2	37	0.000
	Suspicion of depressive disorders identified	20	121	
	T1 not administered	10	0	

**Table 9:** Pearson correlations Between T0 values and CDI.

Correlations													
		T0 = General Affected state/Negative Mood	T0 = Problems in Personal life/Family Life	T0 = Inefficiency (as Perceived by the Patient = Work, Study, etc.)	T0 = Low Self-Esteem	T0 = Irritability	T0 = Fatigability	T0 = Insomnia	T0 = Decreased Appetite	T0 = Vices/Substance Use (Alcohol, Tobacco, Drugs)	T0 = Introversion (Whether the Patient Is Introverted or Has Difficulty Initiating/Maintaining Conversations: Eye Contact)	T0 Conclusion	Clinical Significance of CDI
T0 Conclusion	Pearson Correlation	0.129	0.783 **	0.081	0.194 **	0.241 **	0.213 **	0.209 **	0.208 **	0.203 **	0.208 **	1	0.149 *
	Sig. (2-tailed)	0.076	0.000	0.266	0.007	0.001	0.003	0.004	0.004	0.005	0.004		0.040
	N	190	190	190	190	190	190	190	190	190	190	190	190
Clinical significance of CDI	Pearson Correlation	−0.187 **	0.149 *	0.162 *	0.085	0.097	0.168 *	0.171 *	0.169 *	0.171 *	0.169 *	0.149 *	1
	Sig. (2-tailed)	0.01	0.041	0.026	0.243	0.183	0.020	0.019	0.019	0.018	0.019	0.040	
	N	190	190	190	190	190	190	190	190	190	190	190	190

* Correlation is a significant at the 0.05 level (2-tailed) ** Correlation is a significant at the 0.01 level (2-tailed).

**Table 10:** Pearson correlations between T1 and CDI.

Correlations													
		T1 = General Affected State/Negative Mood	T1 = Problems in Personal Life/Family Life	T1 = Inefficiency (as Perceived by the Patient = Work, Study, etc.)	T1 = Low Self-Esteem	T1 = Irritability	T1 = Fatigability	T1 = Insomnia	T1 = Decreased Appetite	T1 = Vices/Substance Use (Alcohol, Tobacco, Drugs)	T1 = Introversion (Whether the Patient Is Introverted or Has Difficulty Initiating/Maintaining Conversations: Eye Contact)	T1 Conclusion	Clinical Significance of CDI
T1 Conclusion	Pearson Correlation	0.218 **	0.245 **	0.421 **	0.229 **	0.178 **	0.252 **	0.258 **	0.145	0.039	0.188 *	1	0.441 **
	Sig. (2-tailed)	0.003	0.001	0.000	0.002	0.017	0.001	0.000	0.052	0.600	0.011		0.000
	N	180	180	180	180	180	180	180	180	180	180	190	190
Clinical significance of CDI	Pearson Correlation	0.036	0.149 *	0.044	0.114	−0.039	−0.025	−0.006	0.242 **	0.164 *	−0.112	0.441 **	1
	Sig. (2-tailed)	0.632	0.046	0.553	0.126	0.605	0.741	0.938	0.001	0.028	0.135	0.000	
	N	180	180	180	180	180	180	180	180	180	180	190	190

* Correlation is a significant at the 0.05 level (2-tailed) ** Correlation is a significant at the 0.01 level (2-tailed).

**Table 11:** Synoptic table with the distribution of patients in the study group dependent on the responses obtained at T0 and T1.

		T1 = Affected General Condition/Negative Mood		T1 = Problems In Personal Life/Family Life		T1 = Inefficiency (as Considered by the Patient = Work, Learning, Etc.)		T1 = Low Self-Esteem		T1 = Irritability		T1 = Fatigue		T1 = Insomnia		T1 = Decreased Appetite		T1 = Vices/Substance Use (Alcohol, Tobacco, Drugs)		T1 = Introversion (If the Patient Is an Introvert, or If They Have the Ability to Initiate/Have Dialogs, Looking in the Eyes)		Conclusion T1		
		No	Yes	No	Yes	No	Yes	No	Yes	No	Yes	No	Yes	No	Yes	No	Yes	No	Yes	No	Yes	There Is No Suspicion of Occurrence of Depressive Disorders	The Suspicion of Depressive Disorders Is Identified	T1 Was Not Performed
T0 = affected general condition/negative mood	No	4	30	30	4	24	10	9	25	20	14	15	19	29	5	11	23	34	0	12	22	13	21	6
	Yes	9	137	87	59	61	85	47	99	79	67	58	88	75	71	66	80	145	1	30	116	26	120	4
T0 = problems in personal life/family life	No	1	14	12	3	7	8	4	11	10	5	5	10	12	3	13	2	15	0	11	4	5	10	1
	Yes	12	153	105	60	78	87	52	113	89	76	68	97	92	73	64	101	164	1	31	134	34	131	9
T0 = inefficiency (as considered by the patient = work, learning, etc.)	No	5	89	80	14	42	52	28	66	56	38	33	61	48	46	39	55	93	1	19	75	20	74	3
	Yes	8	78	37	49	43	43	28	58	43	43	40	46	56	30	38	48	86	0	23	63	19	67	7
T0 = low self-esteem	No	2	47	32	17	38	11	20	29	27	22	17	32	24	25	23	26	48	1	14	35	16	33	2
	Yes	11	120	85	46	47	84	36	95	72	59	56	75	80	51	54	77	131	0	28	103	23	108	8
T0 = irritability	No	2	27	16	13	16	13	17	12	13	16	9	20	13	16	15	14	29	0	10	19	7	22	1
	Yes	11	140	101	50	69	82	39	112	86	65	64	87	91	60	62	89	150	1	32	119	32	119	9
T0 = fatigue	No	5	73	55	23	32	46	23	55	57	21	31	47	44	34	35	43	78	0	13	65	17	61	2
	Yes	8	94	62	40	53	49	33	69	42	60	42	60	60	42	42	60	101	1	29	73	22	80	8
T0 = insomnia	No	5	74	56	23	33	46	23	56	58	21	32	47	45	34	35	44	79	0	13	66	18	61	2
	Yes	8	93	61	40	52	49	33	68	41	60	41	60	59	42	42	59	100	1	29	72	21	80	8
T0 = decreased appetite	No	3	52	32	23	22	33	13	42	31	24	23	32	41	14	30	25	55	0	14	41	14	41	1
	Yes	10	115	85	40	63	62	43	82	68	57	50	75	63	62	47	78	124	1	28	97	25	100	9
T0 = vices/substance use (alcohol, tobacco, drugs)	No	3	53	33	23	23	33	13	43	32	24	24	32	42	14	30	26	56	0	14	42	15	41	1
	Yes	10	114	84	40	62	62	43	81	67	57	49	75	62	62	47	77	123	1	28	96	24	100	9
T0 = introversion (if the patient is an introvert, or if they has the ability to initiate/have dialogs, looking in the eyes)	No	3	52	32	23	22	33	13	42	31	24	23	32	41	14	30	25	55	0	14	41	14	41	1
	Yes	10	115	85	40	63	62	43	82	68	57	50	75	63	62	47	78	124	1	28	97	25	100	9
Conclusion T0	There is no suspicion of depressive disorders	1	17	15	3	10	8	7	11	11	7	6	12	14	4	12	6	18	0	10	8	6	12	1
	The suspicion of depressive disorders is identified	12	150	102	60	75	87	49	113	88	74	67	95	90	72	65	97	161	1	32	130	33	129	9

**Table 12:** Synoptic table of the results obtained during the CDI.

				Decision to Carry Out the CDI		Clinical Significance of CDI		
				It Was Not Performed	It Was Performed	No Clinically Significant Values	The Values Obtained Are of Clinical Significance	No CDI Performed Conducted
Conclusion T0	There is no suspicion of depressive disorders			1	18	3	15	1
	The suspicion of depressive disorders is identified			31	140	9	131	31
Conclusion T1	There is no suspicion of depressive disorders			2	37	8	29	2
	The suspicion of depressive disorders is identified			20	121	4	117	20
	T1 was not performed			10	0	0	0	10
Conclusion T0	There is no suspicion of depressive disorder occurrence	Conclusion T1	There is no suspicion of depressive disorder occurrence	0	6	1	5	0
			The suspicion of depressive disorder occurrence is identified	0	12	2	10	0
			T1 was not performed	1	0	0	0	1
	The suspicion of depressive disorder occurrence is identified	Conclusion T1	There is no suspicion of depressive disorder occurrence	2	31	7	24	2
			The suspicion of depressive disorder occurrence is identified	20	109	2	107	20
			T1 was not performed	9	0	0	0	9

## Data Availability

The datasets generated and/or analyzed during the current study are not publicly available due ethical reasons but are available from the corresponding author upon reasonable request.
